# Supplementary material for: The Construction and Exploration of a Comprehensive MicroRNA Centered Regulatory Network in Foxtail Millet (Setaria italica L.)
Source: Front Plant Sci. 2022 May 6;13:848474. doi: 10.3389/fpls.2022.848474 (PMC9121102; doi:10.3389/fpls.2022.848474)
Supplement: Supplementary file 5 [file Data_Sheet_5.zip › Sit-miR393b_Seita.4G004900.1_1238_TPlot.pdf]

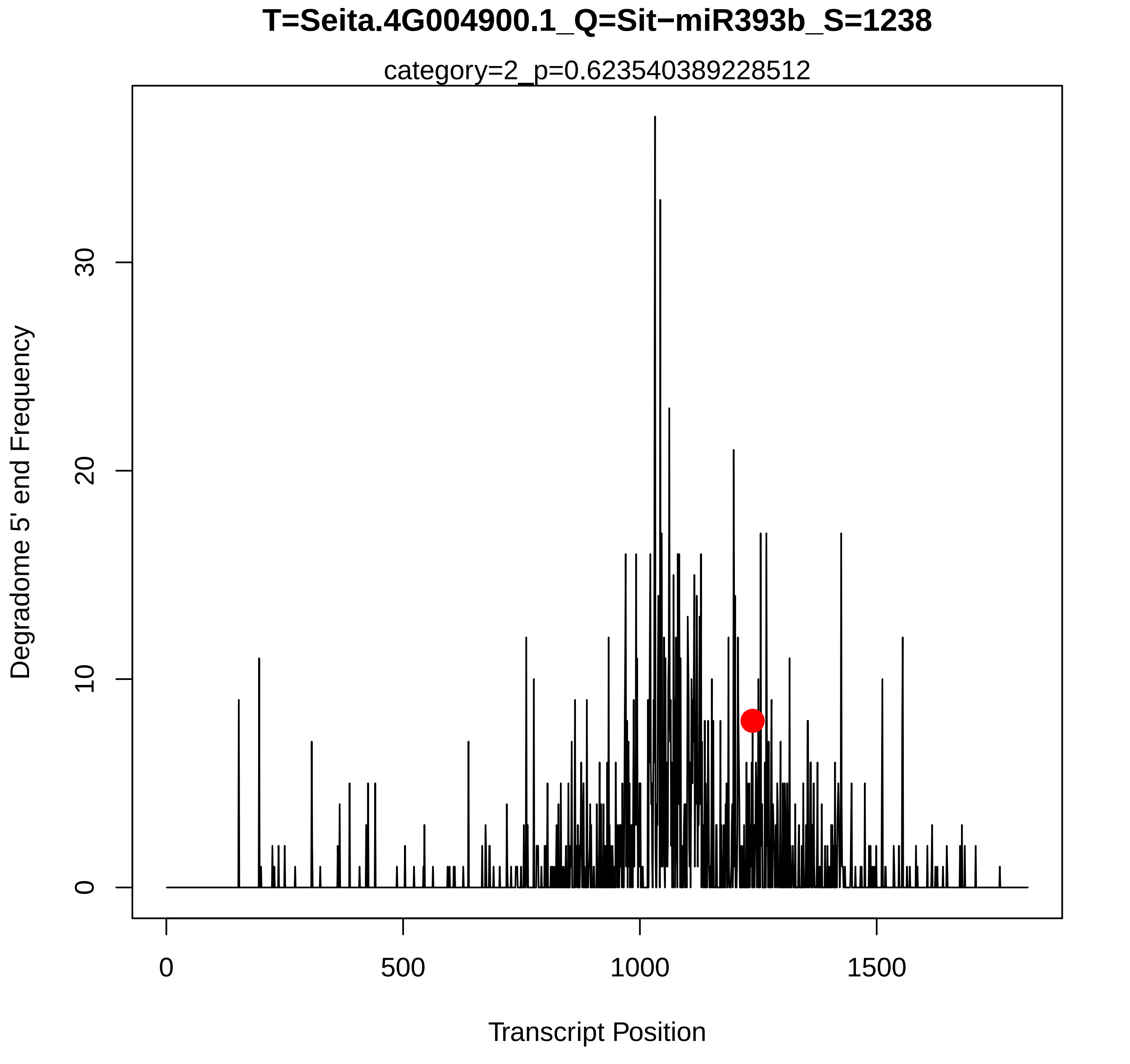
T=Seita.4G004900.1_Q=Sit−miR393b_S=1238
category=2_p=0.623540389228512
Degradome 5' end Frequency
0
10
20
30
0
500
1000
1500
Transcript Position
